# Dual Surveillance of Surgical Site Infections Using CDC and ASEPSIS Criteria: Clinical and Economic Outcomes in Colorectal and Small Bowel Surgery

**DOI:** 10.1002/wjs.70102

**Published:** 2025-09-18

**Authors:** Jung Rae Cho, Duck‐Woo Kim, Myoung Jin Shin, Eu Suk Kim, Hong Bin Kim, Min Hyun Kim, Heung‐Kwon Oh, Sung‐Bum Kang

**Affiliations:** ^1^ Department of Surgery Dankook University College of Medicine Cheonan Korea; ^2^ Department of Surgery Seoul National University Bundang Hospital Seongnam Korea; ^3^ Infection Control Office Seoul National University Bundang Hospital Seongnam Korea; ^4^ Department of Internal Medicine Seoul National University Bundang Hospital Seongnam Korea

**Keywords:** colorectal surgery, costs and cost analysis, surgical wound infection

## Abstract

**Background:**

Surgical site infection (SSI) adversely affects postoperative outcomes and resource utilization. CDC‐based definitions may under‐identify minor yet clinically significant wound healing disturbances. This study evaluated the incidence and burden of SSIs using both the CDC criteria and the ASEPSIS scoring system.

**Methods:**

A prospective observational study was conducted at a tertiary referral center between August 2018 and January 2019. Patients undergoing colorectal or small bowel surgery were monitored for SSIs using CDC criteria and ASEPSIS scores. Surgical wounds were assessed on postoperative days 2, 4, and 6 with follow‐up until 30 days. SSI was defined as either meeting the CDC criteria or having an ASEPSIS score > 21. ASEPSIS scores of 10–19 were classified as wound disturbance.

**Results:**

Among 460 patients, 54 (11.7%) developed SSIs: 53 based on CDC criteria and 15 based on ASEPSIS score. When ASEPSIS scores of 10–19 were included, the wound complication rate increased to 17.6%. Patients with SSIs had longer hospital stays (15.6 ± 8.0 vs. 11.0 ± 5.5 days, *p* < 0.001) and incurred 36% higher total hospital costs. Among patients with ASEPSIS scores of 10–19 but no CDC‐defined SSI (*n* = 28), hospital stay and inpatient costs were comparable to those of SSI‐free patients. However, outpatient visits (6.5 ± 9.3 vs. 1.1 ± 1.3, *p* = 0.005) and costs (USD 99 ± 91 vs. USD 48 ± 48, *p* = 0.007) were significantly higher.

**Conclusions:**

The ASEPSIS scoring system identified clinically relevant wound disturbances that were not detected by CDC criteria. Dual surveillance revealed additional postoperative morbidity and outpatient burden. Integrating ASEPSIS into routine monitoring may improve wound complication detection and enable earlier interventions.

## Graphical abstract

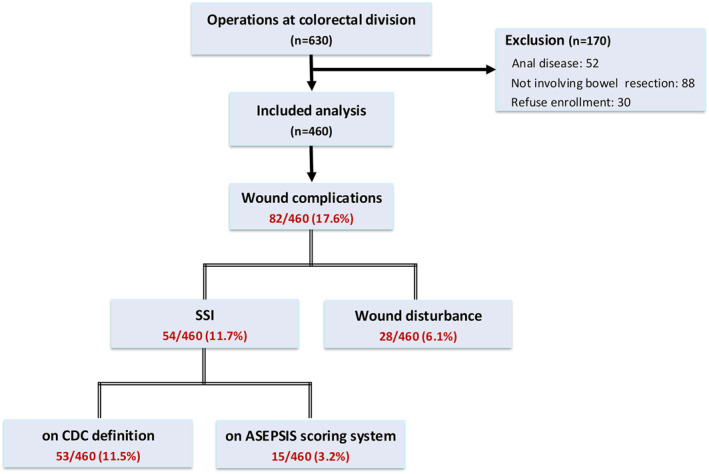

## 1 Introduction

Surgical site infection (SSI) is the leading cause of nosocomial infections following surgery. Various surgical outcomes, including length of hospital stay, overall patient satisfaction, medical expenses, and oncologic outcomes, are adversely affected by SSIs [1]. According to the Korean national healthcare‐associated infections surveillance system, a nationwide multicenter web‐based database, SSI rates range from 3.0% to 4.3% in patients undergoing colonic surgery and from 1.8% to 5.8% in those undergoing rectal surgery [2, 3]. These estimates are lower than those reported in other international studies, where SSI rates ranged from 3.6% to 45.0% [4, 5, 6, 7, 8, 9, 10].

The Centers for Disease Control and Prevention (CDC) criteria [11] are the commonly used definitions for SSIs. However, they rely heavily on subjective clinical judgment, such as the interpretation of wound drainage or local signs of inflammation, and are variably applied across institutions [12]. These criteria may under‐identify minor yet clinically relevant wound disturbances that do not meet the diagnostic threshold for an SSI but still require medical intervention. Such subclinical wound complications may contribute to delayed discharge, increased outpatient follow‐up, and higher medical costs, yet they are often overlooked in formal surveillance systems. Another wound scoring system, the additional treatment, serous discharge, erythema, purulent exudate, separation of deep tissues, isolation of bacteria, and duration of inpatient stay (ASEPSIS) score, offers a structured point‐based evaluation of postoperative wound healing. Originally designed for use in cardiac surgery [13], the ASEPSIS score quantifies wound characteristics, such as erythema, exudate, and separation, and assigns additional points for clinical interventions, such as antibiotic use or debridement. This score may provide a quantitative measure of the severity of wound disturbance [14]. Although it does not replace the CDC definition, the ASEPSIS criteria may complement existing surveillance systems by identifying wound‐related complications that would otherwise go unreported. Despite these theoretical advantages, limited data exist on how combining CDC and ASEPSIS criteria might provide a broader picture of wound‐related complications following gastrointestinal surgery. Moreover, the clinical and economic significance of these additional wound disturbances remains underexplored in real‐world settings. Therefore, we conducted a prospective observational study to assess the incidence and clinical burden of surgical site complications following colorectal and small bowel surgery by concurrently applying both the CDC criteria and the ASEPSIS scoring system. This dual‐assessment strategy aimed to provide a more comprehensive understanding of postoperative wound complications, including those that may be underreported by standard definitions.

## 2 Materials and Methods

### 2.1 Patients and Treatments

This prospective observational study was conducted to identify SSI in consecutive patients who underwent surgical procedures performed by five surgeons in the colorectal division of a high‐volume tertiary referral center between August 2018 and January 2019. Patients were excluded if they underwent surgery for anal diseases, had procedures using a transanal approach (e.g., transanal local excision for rectal cancer), or underwent surgery without bowel resection (e.g., adhesiolysis, mass excision, or hernia repair). The study protocol was approved by the relevant institutional ethical review board (B‐1809‐495‐301), and all patients provided written informed consent to participate in the study. This study adheres to the Strengthening the Reporting of Observational Studies in Epidemiology (STROBE) guidelines. A completed STROBE checklist is included in Table S1.

### 2.2 Evaluation of Surgical Wounds

All patients underwent a routine SSI prevention bundle, including mechanical bowel preparation, parenteral antibiotic prophylaxis using second‐generation cephalosporin prior to skin incision, intraoperative skin disinfection using 7% betadine and 2% chlorhexidine in 70% alcohol, glove changes after each intraoperative digital rectal exam, wound irrigation with saline followed by closure using interrupted sutures and staples, and dressing changes 48 h postoperatively.

SSIs were assessed using both the conventional CDC criteria and the ASEPSIS scoring system (Table 1) during postoperative wound dressing. Wound status was evaluated on postoperative days 2, 4, and 6 with photodocumentation for further review by a second physician. Patients were followed up for SSI occurrence for 30 days postoperatively or until complete wound healing. An SSI was diagnosed if the wound met the CDC criteria or had an ASEPSIS score exceeding 21 points on the ASEPSIS score, indicating a minor or more severe infection. That is, patients were classified as having SSI if either of the two criteria was met. In cases where the CDC and ASEPSIS assessments were discordant, the presence of either a CDC‐defined SSI or an ASEPSIS score > 21 was considered sufficient for inclusion as an SSI case. Among these, only one patient fulfilled the ASEPSIS criteria but did not meet the CDC definition.

**TABLE 1:** Definitions of SSI used in this study.

CDC criteria [11]
Superficial incisional SSI Infection limited to the skin or subcutaneous tissue occurring within 30 days postoperatively, meeting at least one of the following conditions: Purulent discharge from the superficial incision, regardless of laboratory confirmation. Isolation of organisms from a culture obtained aseptically from fluid or tissue at the superficial incision site. At least one local sign or symptom, such as pain, swelling, redness, or heating sense, combined with intentional reopening of the incision by a surgeon, unless the culture is negative. Clinical diagnosis of superficial SSI made by the operating surgeon or attending physician. Deep incisional SSI Infection involving the deep soft tissues (e.g., fascia or muscle) of the incision within 30 days after the operation, with evidence suggesting it is procedure‐related, and fulfilling at least one of the following: Purulent drainage originating from the deep portion of the incision, without involvement of organ/space structures. Dehiscence or surgical reopening of the deep incision in the presence of fever (> 38°C), localized pain, or tenderness, unless a negative culture is obtained. Detection of abscess or infection within the deep incision during clinical examination, reoperation, or via histopathological or imaging studies. Clinical diagnosis of deep incisional SSI by a surgeon or the attending physician.

ASEPSIS score [13]					
Wound characteristics	Proportion of wounds affected (%)				
	≤ 20	20–39	40–59	60–79	≥ 80
Serous exudate	1	2	3	4	5
Erythema	1	2	3	4	5
Purulent exudate	2	4	6	8	10
Separation of deep tissue	2	4	6	8	10
Additional points					
Antibiotics	10				
Drainage at bedside	5				
Debridement at operation room	10				
Isolation of bacteria	10				

*Note:* The ASEPSIS score is calculated as the sum of points assigned for each wound characteristic and intervention. Based on the total score, wound outcomes are categorized as follows: 0−9, satisfactory; 10−19, disturbance of healing; 20−29, minor SSI; 30−39, moderate SSI; ≥ 40, severe SSI.

Abbreviation: SSI, Surgical Site Infection.

### 2.3 Outcomes and Data Collection

The primary outcome was the incidence of SSI, defined as the presence of infection based on either the CDC criteria or an ASEPSIS score > 21 following major colorectal or small bowel surgery. Secondary outcomes included clinical and economic parameters such as hospital stay, inpatient and outpatient costs, and the number of outpatient clinic visits. In addition to patients with SSIs, a subgroup of patients with ASEPSIS scores of 10–19, which did not meet CDC or ASEPSIS SSI thresholds, was classified as having wound healing disturbance. This wound disturbance group was included in secondary analyses due to its potential clinical relevance. Patient demographic characteristics and perioperative variables relevant to SSI development and management were prospectively obtained from electronic medical records, which incorporated both patient‐reported information and physician‐prescribed medications.

### 2.4 Statistical Analysis

Continuous variables were compared using either Student's *t*‐test or Mann–Whitney *U* test, whereas categorical variables were compared using chi‐square or Fisher's exact test. Univariate logistic regression analysis was performed to identify risk factors for SSI development. Variables with a *p* value < 0.1 in the univariate analysis were included in the multivariate logistic regression analysis. All statistical analyses were performed using IBM SPSS statistics 25.0 system (IBM, Armonk, New York, United States), with *p* < 0.05 considered statistically significant. Subgroup analyses were conducted to compare clinical and economic outcomes among patients with in‐hospital SSIs, post‐discharge SSIs, wound healing disturbances, and those without any wound complications. All enrolled patients completed follow‐up without missing data for primary outcomes or key variables; therefore, imputation was not necessary, and sensitivity analyses were not performed.

## 3 Results

### 3.1 Patient Enrollment and SSI Incidence

**FIGURE 1:**
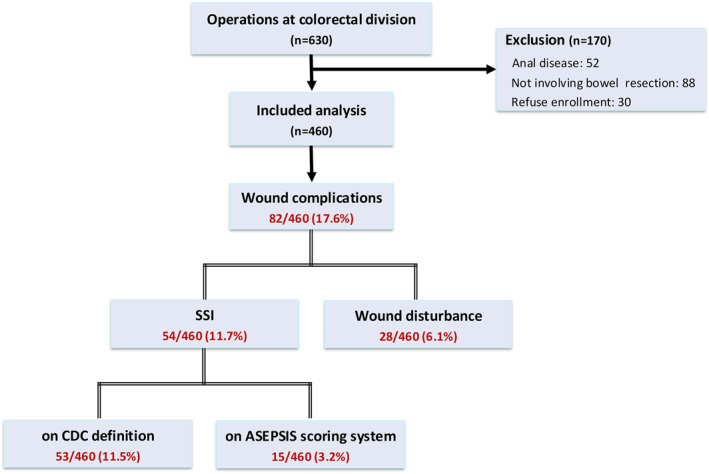
Flow diagram of the study patients.

During the study period, 630 patients underwent surgery under the colorectal division. Among them, 170 were excluded due to anal disease or transanal approach (*n* = 52), surgery without bowel resection (*n* = 88), and refusal to participate (*n* = 30). Of the 460 patients included in the final analysis, 54 (11.7%) developed postoperative SSIs. Based on diagnostic criteria, 53 patients met the CDC definition for SSI, and 15 patients had an ASEPSIS score > 21 (Figure 1). Among the CDC‐positive group, 45 patients (85.0%) were classified as having superficial incisional SSIs, 4 (7.5%) as deep incisional, and 4 (7.5%) as organ/space SSIs. Among the 15 patients classified as having SSI by ASEPSIS criteria, 12 (80.0%) were classified as having minor SSI (score 20–29), and 3 (20.0%) as moderate SSI (score 30–39). When *patients with wound healing disturbances* were included, the overall rate of wound‐related complications increased to 17.6% (82/460). Representative photographs corresponding to ASEPSIS scores are shown in Figure S1.

### 3.2 Risk Factors for SSI

Several factors were associated with the development of SSIs (Table 2). SSI incidence was significantly higher in contaminated wounds compared to clean‐contaminated wounds (40.0% vs. 10.8%, *p* < 0.001) and in colorectal surgery compared to small bowel surgery (13.3% vs. 6.6%, *p* = 0.061). In the multivariate logistic regression analysis, contaminated wound classification remained a significant risk factor (odds ratio [OR] 3.86, 95% confidence interval [CI] 1.20–12.38, *p* = 0.023), whereas small bowel surgery (vs. colorectal) was associated with lower SSI risk (OR 0.31, 95% CI 0.12–0.83, *p* = 0.020). Other factors, including serum albumin level (OR 0.64, 95% CI 0.37–1.10, *p* = 0.105) and laparoscopic approach (OR 0.46, 95% CI 0.21–1.03, *p* = 0.058), did not reach statistical significance.

**TABLE 2:** Baseline characteristics and uni‐ and multivariate analyses of risk factors for SSI.

Characteristics	Univariate analysis			Multivariate analysis	
	SSI‐detected (*N* = 54)	SSI‐free (*N* = 406)	*p* value	OR (95% CI)	*p* value
Age (years)	59.8 ± 16.0	62.4 ± 13.8	0.193		
Sex			0.721		
Male	35 (64.8)	253 (62.3)			
Female	19 (35.2)	153 (37.7)			
BMI	23.3 ± 4.1	23.4 ± 3.5	0.823		
ASA class			0.250		
1, 2	47 (87.0)	327 (80.5)			
≥ 3	7 (13.0)	79 (19.5)			
Heart disease	1 (1.9)	27 (6.8)	0.170		
Anticoagulation	6 (11.5)	36 (9.1)	0.569		
Liver disease	1 (1.9)	9 (2.3)	0.871		
Hypertension	21 (38.9)	162 (39.9)	0.886		
DM	10 (18.5)	102 (25.2)	0.280		
Current smoker^a^	8 (15.4)	47 (11.9)	0.468		
Malignancy	46 (85.2)	315 (77.6)	0.202		
Wound classification			< 0.001		
Clean‐contaminated	48 (88.9)	397 (97.8)		1 (Ref)	
Contaminated	6 (11.1)	9 (2.2)		3.859 (1.203–12.383)	0.023
Emergency operation	10 (18.5)	52 (12.8)	0.248		
Reoperation < 30 days	1 (1.9)	3 (0.7)	0.408		
Preoperative functioning ostomy			0.454		
None	47 (87.0)	337 (83.)			
Ileostomy or colostomy	7 (13.0)	69 (17.0)			
Preoperative laboratory findings					
WBC	7.4 ± 2.9	7.0 ± 3.0	0.454		
Hemoglobin	12.3 ± 1.8	12.7 ± 2.0	0.128		
Albumin	3.9 ± 0.6	4.1 ± 0.5	0.014	0.639 (0.372–1.098)	0.105
BST	111.0 ± 31.8	116.1 ± 34.1	0.300		
HbA1c	5.9 ± 1.2	6.0 ± 1.1	0.558		
CRP	4.4 ± 7.4	3.4 ± 5.4	0.447		
Perioperative findings					
Operation type			0.061		
Colectomy, proctectomy	47 (87.0)	307 (75.6)		1 (Ref)	
Ileostomy repair, other SB operation, and appendectomy	7 (13.0)	99 (24.4)		0.309 (0.115–0.832)	0.020
Operation team size	3.5 ± 0.9	3.4 ± 0.8	0.186		
Operation time	183.0 ± 85.9	157.5 ± 76.1	0.024	1.002 (0.999–1.006)	0.206
Approach method			0.004		
Open	23 (42.6)	98 (24.1)		1 (Ref)	
Laparoscopy	31 (57.4)	308 (75.9)		0.463 (0.209–1.026)	0.058
Ostomy creation			0.380		
None	37 (68.5)	301 (74.1)			
Ileostomy or colostomy	17 (31.5)	105 (25.9)			
Wound drain in situ	13 (24.1)	80 (19.7)	0.453		
Wound protector application	23 (42.6)	229 (56.4)	0.055	0.623 (0.305–1.274)	0.195
Co‐operation	8 (14.8)	53 (13.1)	0.720		

*Note:* Continuous variables are expressed as mean ± standard deviation; categorical variables are expressed as numbers (%) of patients.

Abbreviations: ASA, American Society of Anesthesiologists; BMI, Body Mass Index; BST, Blood Sugar Test; CI, Confidence Interval; CRP, C‐Reactive Protein; DM, Diabetes Mellitus; HbA1c, Hemoglobin A1c; OR, Odds ratio; SB, Small Bowel; SSI, Surgical Site Infection; WBC, White Blood Cells.

^a^ Cessation of smoking 2 weeks prior to surgery was excluded.

### 3.3 Clinical and Economic Impact of SSI

Table 3 summarizes the clinical and economic impact of SSIs. Patients with SSIs diagnosed during hospitalization had a median hospital stay of 19 days (interquartile range (IQR): 16–27), nearly twice that of patients without SSIs (10 days; IQR: 8–12; *p* < 0.001). In contrast, patients with SSIs detected after discharge had a comparable length of stay to those without SSIs (10 days; IQR: 9–11; *p* = 0.749). Surgical wound repair was performed significantly more often in patients with in‐hospital SSIs than in those with post‐discharge SSIs (84.6% vs. 25.0%, *p* < 0.001).

**TABLE 3:** Clinical impact of SSI.

	SSI‐detected			SSI‐free (*N* = 406)	*p* value^b^
	During hospitalization (*N* = 26)	After discharge (*N* = 28)	*p* value^a^		
Length of hospital stay (days)	19 (16–27)	10 (9–11)	< 0.001	10 (8–12)	< 0.001
Readmission	3 (11.5)	6 (21.4)	0.470		
Usage of antibiotics	13 (50.0)	8 (28.6)	0.163		
Surgical wound repair	22 (84.6)	7 (25.0)	< 0.001		
Total hospitalization cost (USD)	13,958 (10,447–16,226)	8998 (7091–11,042)	< 0.001	8604 (6619–10,467)	< 0.001
Total outpatient clinic cost (USD)	19 (5–74)	149 (110–266)	< 0.001	38 (19–70)	< 0.001
Number of outpatient clinic visits	3 (1–5)	6 (4–8)	0.003	1 (0–2)	< 0.001

*Note:* Continuous variables are expressed as median (IQR); categorical variables are expressed as numbers (%) of patients.

Abbreviations: IQR, interquartile range; SSI, Surgical Site Infection.

^a^ Compared between SSI detected during hospitalization and after discharge.

^b^ Compared between the SSI‐detected and SSI‐free group.

Patients with in‐hospital SSIs had significantly higher treatment costs than those without SSIs (median: USD 13,958; IQR: 10,447–16,226 vs. USD 8604; IQR 6619‐10,467; *p* < 0.001). In contrast, the costs associated with post‐discharge SSIs did not differ significantly from those without SSIs (median: USD 8998; IQR: 7091–11,042; *p* = 0.186). Outpatient burden was significantly higher in the post‐discharge SSI group than in the SSI‐free group, with increased outpatient clinic costs (median: USD 149; IQR: 110–266 vs. USD 38; IQR: 19–70; *p* < 0.001) and a greater number of visits (median: 6; IQR: 4–8 vs. 1; IQR: 0–2; *p* = 0.003).

Total hospitalization costs were lower for the 28 patients with ASEPSIS scores of 10–19, classified as having wound healing disturbances without meeting the SSI criteria, than for those with SSIs (median: USD 9470; IQR: 7084–11,002 vs. USD 10,951; IQR: 8563–13,958; *p* = 0.014). Compared to SSI‐free patients, the wound disturbance group exhibited a higher number of outpatient visits (median 3; IQR: 1–8 vs. 1; IQR: 0–2; *p* < 0.001) and greater outpatient‐related expenses (median: USD 68; IQR: 21–144 vs. USD 37; IQR: 19–69; *p* = 0.011) with no significant difference in in‐hospital stay and cost (Table 4).

**TABLE 4:** Clinical impact of wound healing disturbance, not diagnosed by SSI.

	SSI‐detected (*N* = 54)	Wound disturbance (*N* = 28)	*p* value^a^	SSI‐free (*N* = 378)	*p* value^b^
Length of hospital stay (days)	12 (10–18)	11 (8–14)	0.062	10 (8–12)	0.065
Readmission	9 (16.7)	0	0.025		
Usage of antibiotics	21 (38.9)	2 (7.1)	0.002		
Surgical wound repair	29 (53.7)	5 (17.9)	0.002		
Total hospitalization cost (USD)	10,951 (8563–13,958)	9470 (7084–11,002)	0.014	8572 (6601–10,419)	0.431
Total outpatient clinic cost (USD)	107 (20–193)	68 (21–144)	0.539	37 (19–69)	0.011
Number of outpatient clinic visits	5 (2–7)	3 (1–8)	0.424	1 (0–2)	< 0.001

*Note:* Continuous variables are expressed as the median (IQR); categorical variables are expressed as numbers (%) of patients.

Abbreviations: IQR, interquartile range; SSI, Surgical Site Infection.

^a^ Compared between wound disturbance and SSI detected according to the CDC criteria or ASEPSIS scores over 20.

^b^ Compared between wound disturbance and SSI‐free group.

## 4 Discussion

This prospective study evaluated the incidence, clinical characteristics, and economic burden of SSIs following colorectal or small bowel surgery using both the CDC criteria and the ASEPSIS scoring system. The overall SSI incidence was 11.7%; using the CDC criteria alone, the rate was 11.5%, whereas the ASEPSIS score identified SSIs in only 3.2% of patients. When all cases with an ASEPSIS score > 10 were included, comprising both formal SSIs and wound disturbances not classified as infection by traditional definitions, the incidence of wound‐related complications increased to 17.6%. These findings underscore the clinical relevance of wound disturbances detected by the ASEPSIS scoring system, even when they do not meet CDC‐defined SSI criteria.

In our study, 28 patients who were CDC‐negative but classified as having wound healing disturbances based on ASEPSIS scores of 10–19 demonstrated significantly greater outpatient resource utilization, including more frequent clinic visits and higher outpatient costs, compared to those without any wound complications. It is worth noting that wound‐related interventions, such as surgical revision and antibiotic administration, were required in this group alone, despite the absence of criteria‐defined SSI. Although their inpatient outcomes, such as hospital stay and total hospitalization cost, did not differ significantly from those of SSI‐free patients, these wound disturbances were clearly not clinically insignificant. By identifying visible signs of impaired healing and quantifying related interventions, the ASEPSIS scoring system adds granularity to postoperative wound assessment. These findings underscore the need to incorporate ASEPSIS‐based monitoring alongside traditional definitions, particularly in high‐risk surgical populations, as it enables earlier recognition and management of wound‐related morbidity that would otherwise go unnoticed yet contribute significantly to overall patient burden and healthcare resource utilization.

The reported prevalence of SSI following colorectal surgery ranges from 1.8% to 45.0% [2, 3, 4, 5, 6, 7, 8, 9, 10]. This discrepancy highlights a fundamental limitation in SSI surveillance: the choice of diagnostic criteria significantly impacts reported incidence rates. Although widely used, the CDC criteria rely on subjective clinical judgment and are susceptible to variability in institutional reporting, often resulting in under‐ or overestimation of true SSI rates [12, 15, 16]. A Korean study utilizing direct surgeon observation combined with a post‐discharge survey reported an SSI incidence of 10.2% in patients who underwent colectomy and 13.5% in those who underwent proctectomy, based on CDC criteria [17]. These rates are comparable to those of the present study. The active involvement of the surgeon enabled prospective surveillance and may account for the difference in SSI incidence observed in the study compared to nationwide surveillance data in Korea, which reported rates ranging from 1.8% to 5.8% [2, 3]. SSI diagnosis based on the CDC criteria can be subjective due to factors, such as physician reluctance to report SSIs or varying perceptions of SSIs among surgeons or infection control physicians [14, 16].

In contrast, the ASEPSIS score offers a more standardized and objective approach based on observable wound features and clinical interventions [14]. In our study, 28 patients who were not diagnosed with SSI but classified as having wound healing disturbance based on ASEPSIS scores of 10–19 demonstrated significantly greater outpatient resource utilization, including more frequent clinic visits and higher outpatient costs, compared with those without any wound complications. This suggests that even minor wound disturbances have significant clinical consequences. This finding aligns with previous studies suggesting that early or localized wound complications may impair recovery or increase the risk of further complications if not properly managed [18, 19, 20]. Although these cases may resolve with conservative management; nonetheless, they contribute to increased healthcare utilization and patient burden. Importantly, these burdens are often underestimated, as they lie beyond the scope of standard SSI cost analyses. This highlights the utility of the ASEPSIS score in identifying a distinct subgroup of patients with subclinical yet significant wound complications that are often overlooked by conventional SSI surveillance. By identifying observable signs of impaired healing and quantifying associated interventions, the ASEPSIS scoring system adds granularity to postoperative wound assessment. However, the ASEPSIS scoring system is limited to superficial wound assessment and does not detect deep or organ‐space SSIs. This limitation was evident in our study, as some CDC‐defined SSIs were not identified by ASEPSIS score due to their anatomical location or minimal involvement of the superficial wound surface. These findings underscore the need to refine or expand the ASEPSIS criteria to enhance its clinical applicability in abdominal surgery.

Consistent with previous reports, several perioperative factors were associated with an increased risk of SSI. Patients with SSIs were more likely to have contaminated wounds, undergo colorectal rather than small bowel surgery, and receive open rather than laparoscopic procedures. The laparoscopic approach, by reducing tissue trauma and exposure to contamination, has been associated with lower SSI rates [21], and the use of wound protectors may offer additional benefit [22, 23]. Hypoalbuminemia, although not statistically significant in the multivariate analysis, has been previously linked to impaired wound healing and increased infection risk [24, 25]. Diabetes mellitus and smoking have been associated with the development of SSIs [4, 8, 26]. At our institution, perioperative glucose control was optimized in consultation with endocrinology, and patients were advised to cease smoking at least 2 weeks preoperatively to reduce the risk of pulmonary and wound complications [27, 28, 29, 30, 31].

### 4.1 Limitations

This study has several limitations. First, the ASEPSIS scoring system, originally developed for use in cardiac surgery, has not been adequately validated for use in colorectal procedures. Unlike the CDC criteria, which encompass both incisional and intra‐abdominal infections, the ASEPSIS scoring system assesses only incisional infections. Intra‐abdominal complications, such as anastomotic leaks or abscesses, pose greater challenges due to their severity and associated financial burden. Nevertheless, our findings underscore the significant prevalence of incisional wound disturbances and their associated costs, thereby highlighting the need for a focused approach to incisional wound management. Second, the ASEPSIS score is proportional to wound size, potentially misrepresenting the severity of SSI in excessively large or small wounds. Third, the study was conducted at a tertiary hospital, in which an infection control unit actively monitored the quality of patient care. Prospective multicenter studies are required to confirm these findings. Finally, wound culture data were not routinely obtained in the present study.

### 4.2 Conclusions

Among 460 patients undergoing colorectal or small bowel surgery, 11.7% developed SSIs as defined by the CDC criteria or the ASEPSIS scoring system. When subclinical wound disturbances (ASEPSIS scores 10–19) were included, the overall wound‐related complication rate rose to 17.6%, underscoring the need for broader postoperative surveillance. Given its objectivity and ability to identify clinically significant wound disturbances not captured by the CDC criteria, the ASEPSIS scoring system may serve as a valuable adjunct to existing SSI surveillance protocols and warrants integration into routine postoperative wound monitoring, particularly in settings where underreporting may be prevalent.

## Author Contributions

**Jung Rae Cho:** conceptualization, formal analysis, writing – original draft, writing – review and editing. **Duck‐Woo Kim:** conceptualization, formal analysis, funding acquisition, resources, supervision, writing – original draft, writing – review and editing. **Myoung Jin Shin:** resources, writing – review and editing. **Eu Suk Kim:** resources, writing – review and editing. **Hong Bin Kim:** resources, writing – review and editing. **Min Hyun Kim:** resources, writing – review and editing. **Heung‐Kwon Oh:** resources, writing – review and editing. **Sung‐Bum Kang:** resources, writing – review and editing.

## Ethics Statement

The authors comply with the journal's ethical policies. The study protocol was approved by the relevant institutional ethical review board (B‐1809‐495‐301).

## Consent

All patients provided written informed consent to participate in this study.

## Conflicts of Interest

The authors declare no conflicts of interest.

## Supporting information


**Figure S1:** Representative photo‐documented wound according to ASEPSIS score. (a) ASEPSIS score 1, satisfactory wound. (b) ASEPSIS score 10, disturbance of healing. (c) ASEPSIS score 27, minor SSI. (d) ASEPSIS score 32, moderate SSI.

**Table S1:** STROBE Statement: checklist of items that should be included in reports of observational studies.

## Data Availability

The data that support the findings of this study are available on request from the corresponding author. The data are not publicly available due to privacy or ethical restrictions.
