# Supplementary material for: Dual Surveillance of Surgical Site Infections Using CDC and ASEPSIS Criteria: Clinical and Economic Outcomes in Colorectal and Small Bowel Surgery
Source: World J Surg. 2025 Sep 18;49(11):3097–105. doi: 10.1002/wjs.70102 (PMC12582140; doi:10.1002/wjs.70102)

**Supplementary Fig. 1** Representative photo-documented wound according to ASEPSIS score. a, ASEPSIS score 1, satisfactory wound. b, ASEPSIS score 10, disturbance of healing. c, ASEPSIS score 27, minor SSI. d, ASEPSIS score 32, moderate SSI


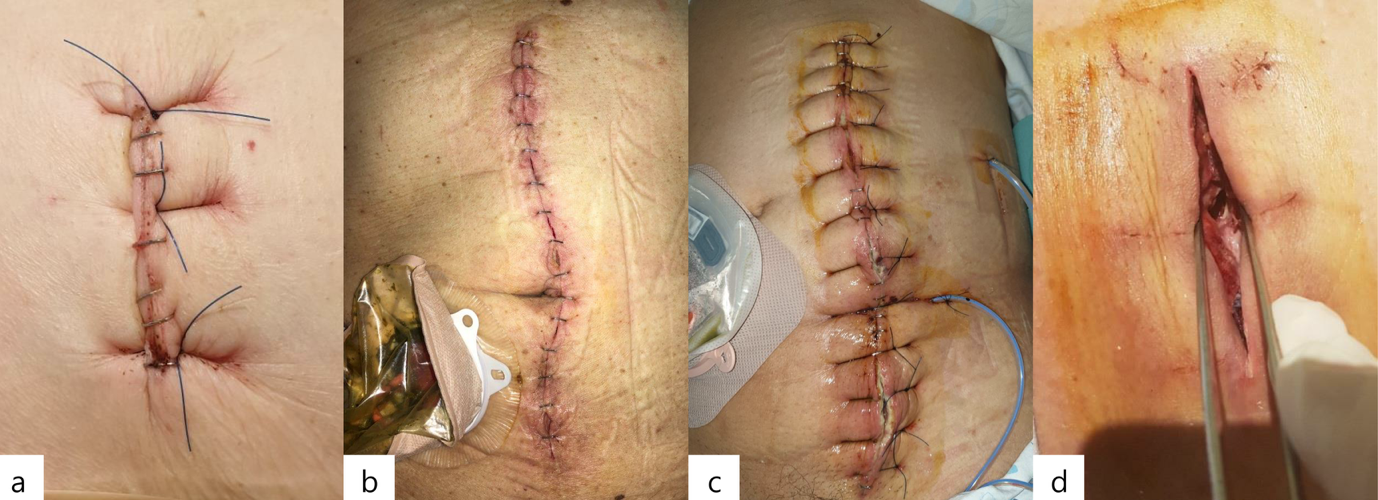

Supplement: Supplementary file 1 — Figure S1: Representative photo‐documented wound according to ASEPSIS score. (a) ASEPSIS score 1, satisfactory wound. (b) ASEPSIS score 10, disturbance of healing. (c) ASEPSIS score 27, minor SSI. (d) ASEPSIS score 32, moderate SSI. [file WJS-49-3097-s002.docx]
